# Thenar Flap: A Workhorse Flap for Fingertip Injuries

**DOI:** 10.7759/cureus.90785

**Published:** 2025-08-23

**Authors:** Vijaykumar Huded, Gazal Gautam

**Affiliations:** 1 General Surgery, BLDE (Deemed to Be University) Shri B. M. Patil Medical College, Hospital and Research Centre, Vijayapura, IND; 2 Hand and Microvascular Surgery, Ganga Hospital, Coimbatore, IND; 3 MCh Plastic and Reconstructive Surgery, Institute of Medical Sciences, Banaras Hindu University, Varanasi, IND

**Keywords:** fingertip injuries, hand injuries, plastic and reconstructive surgery, reconstructive surgical procedure, thenar flap

## Abstract

Introduction: Fingertip injuries are the most common type of upper limb trauma, frequently involving volar oblique or transverse defects with exposed bone or tendon. The thenar flap, first described in 1926, remains a reliable and aesthetically favorable option for reconstruction, particularly for index, middle, and ring finger injuries. Despite concerns about joint stiffness and donor site morbidity, refinements in surgical technique have improved outcomes.

Methods: A retrospective study was conducted at a tertiary care center from January 2023 to January 2025. Twenty-five patients with traumatic fingertip injuries involving pulp loss or volar oblique/transverse defects underwent reconstruction using tailored thenar flap techniques. Functional recovery was assessed via range of motion (ROM) at the metacarpophalangeal joint (MCPJ) and the proximal interphalangeal joint (PIPJ) and static two-point discrimination (2PD). Aesthetic and subjective satisfaction were measured using a five-point Likert scale during a 6-month follow-up.

Results: Among 25 patients (76% male, median age 25 years), the most common injuries were volar oblique (40%) and involved the index finger (56%). Successful flap survival was noted in all cases, with minimal complications. Mean ROM at the MCPJ and PIPJ was 99.92° and 95.36°, respectively. Mean 2PD over reconstructed fingertips was 5.49 mm. Aesthetic satisfaction was high, with 88% of patients rating outcomes as "satisfied" or "very satisfied." Only one patient developed a flexion contracture due to physiotherapy noncompliance.

Conclusion: The thenar flap remains a versatile, safe, and effective option for fingertip reconstruction when performed with proper technique and rehabilitation. It offers excellent functional recovery and aesthetic outcomes, making it a valuable tool across varying age groups.

## Introduction

Fingertip injuries are the most frequent type of upper limb trauma, commonly seen in both industrial settings and household environments. Such injuries can significantly impair fine motor skills and manual dexterity. The thenar flap, first introduced by Gatewood in 1926, remains a well-established and dependable technique for reconstructing complex fingertip injuries, particularly those involving volar defects of the index, middle, and ring fingers [1,2]. This method effectively preserves finger length, sensation, and functionality. Compared to other reconstructive options such as cross-finger flaps, homodigital flaps, and full-thickness skin grafts (FTSG), the thenar flap offers several benefits, including durable, smooth, and hairless skin coverage, sufficient soft tissue cushioning, and excellent color and texture match, which together help restore the fingertip’s natural three-dimensional form [3].

Beasley first described a laterally based thenar flap in 1969, positioning its distal margin at the metacarpophalangeal (MCP) flexion crease of the thumb. He emphasized the importance of proper thumb positioning to reduce flexion at the proximal interphalangeal joint (PIPJ) of the recipient finger [4].

However, the technique is not without drawbacks. Reports in the literature have highlighted complications such as flexion contractures at the proximal interphalangeal joints and noticeable scarring at the donor site. Some even suggest avoiding this method in patients over 30 years of age [5]. Furthermore, technical challenges in flap design, inset, and division, particularly under conditions of tension, rotation, or finger flexion, can make suturing difficult.

This study outlines a refined approach that improves the accuracy and adaptability of the thenar flap design and division, making it more suitable for a broad range of fingertip injuries. The study aims to highlight the unique benefits of this flap technique in fingertip reconstruction and to evaluate both the functional and cosmetic results. This study also aims to evaluate the functional and aesthetic outcomes of the thenar flap in fingertip reconstruction, with a focus on cases involving multiple digits, and to assess its applicability across different age groups.

The objective of this study is to evaluate the outcomes of the thenar flap reconstruction in fingertip injuries. Although more complex options such as microvascular tissue transfer and technically demanding flaps are available, this study does not intend to compare outcomes across different reconstructive techniques. Instead, the emphasis is on the thenar flap, given its relative simplicity, reliability, and suitability in settings where advanced options may not be feasible.

## Materials and methods

Study design and patient selection

This retrospective study was conducted at BLDE (Deemed to Be University) Shri B. M. Patil Medical College, Hospital and Research Centre, Vijayapura, India, from January 2023 to January 2025, following approval from the Institutional Ethics Committee. Patients presenting with fingertip injuries involving multiple digits were included. The primary focus was on reconstruction of the index and middle fingers using the thenar flap, while the remaining injured digits were managed with other appropriate reconstructive procedures. Eligible cases included traumatic fingertip injuries, with or without exposed bone or tendon, involving the index, middle, and ring fingers, irrespective of age or sex. Injuries with transverse or volar oblique defects associated with pulp loss were also included. Written informed consent was obtained from all participants. Patients with injuries over the thenar eminence and those with preexisting joint injuries, arthritis, joint stiffness, or Dupuytren’s contracture were excluded. All patients underwent a standard fingertip injury management protocol and were followed for a period of six months.

Flap design and surgical technique

**Figure 1:**
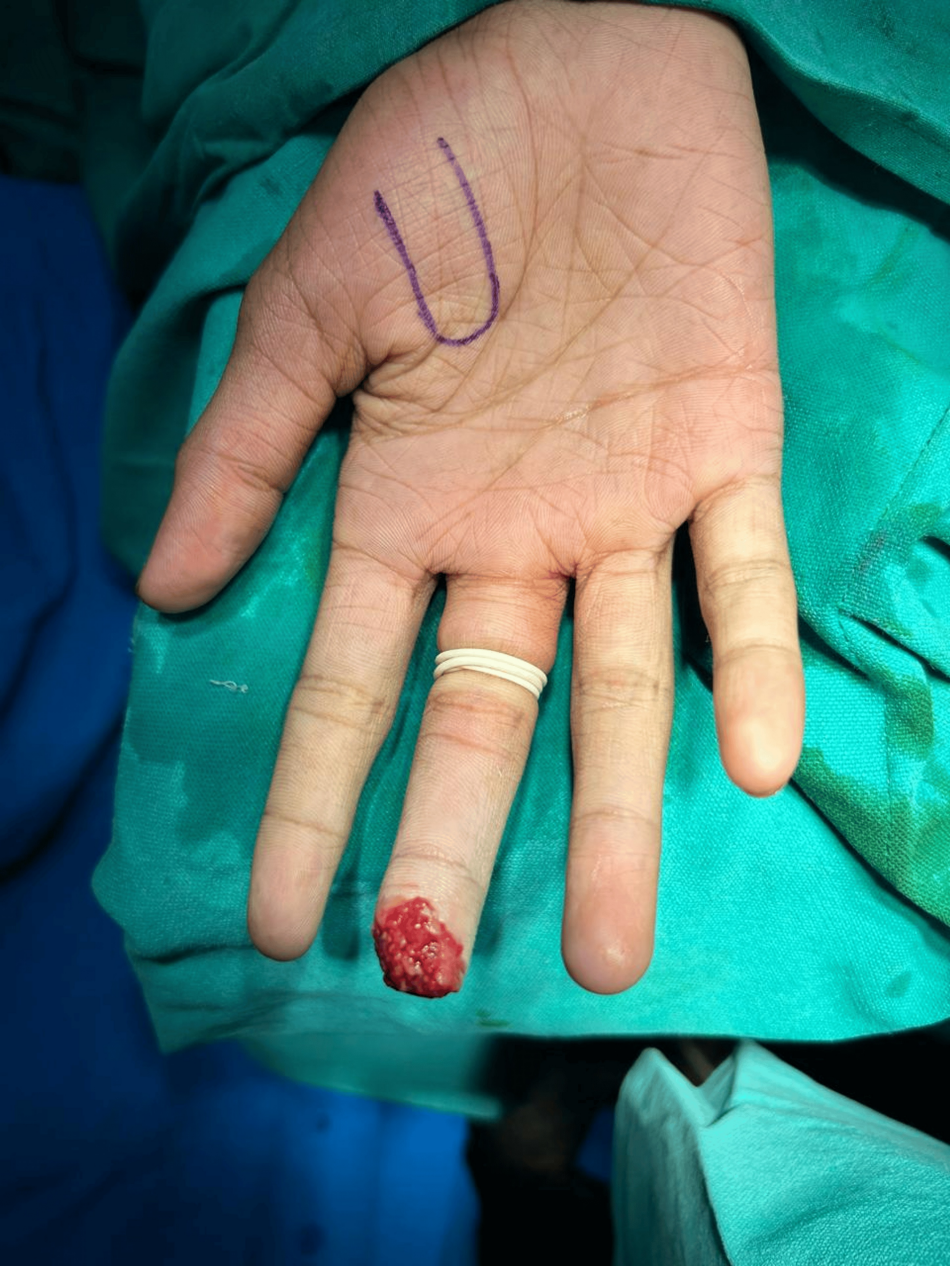
Case 1: Tissue loss of tip of distal phalanx of right third digit

**Figure 2:**
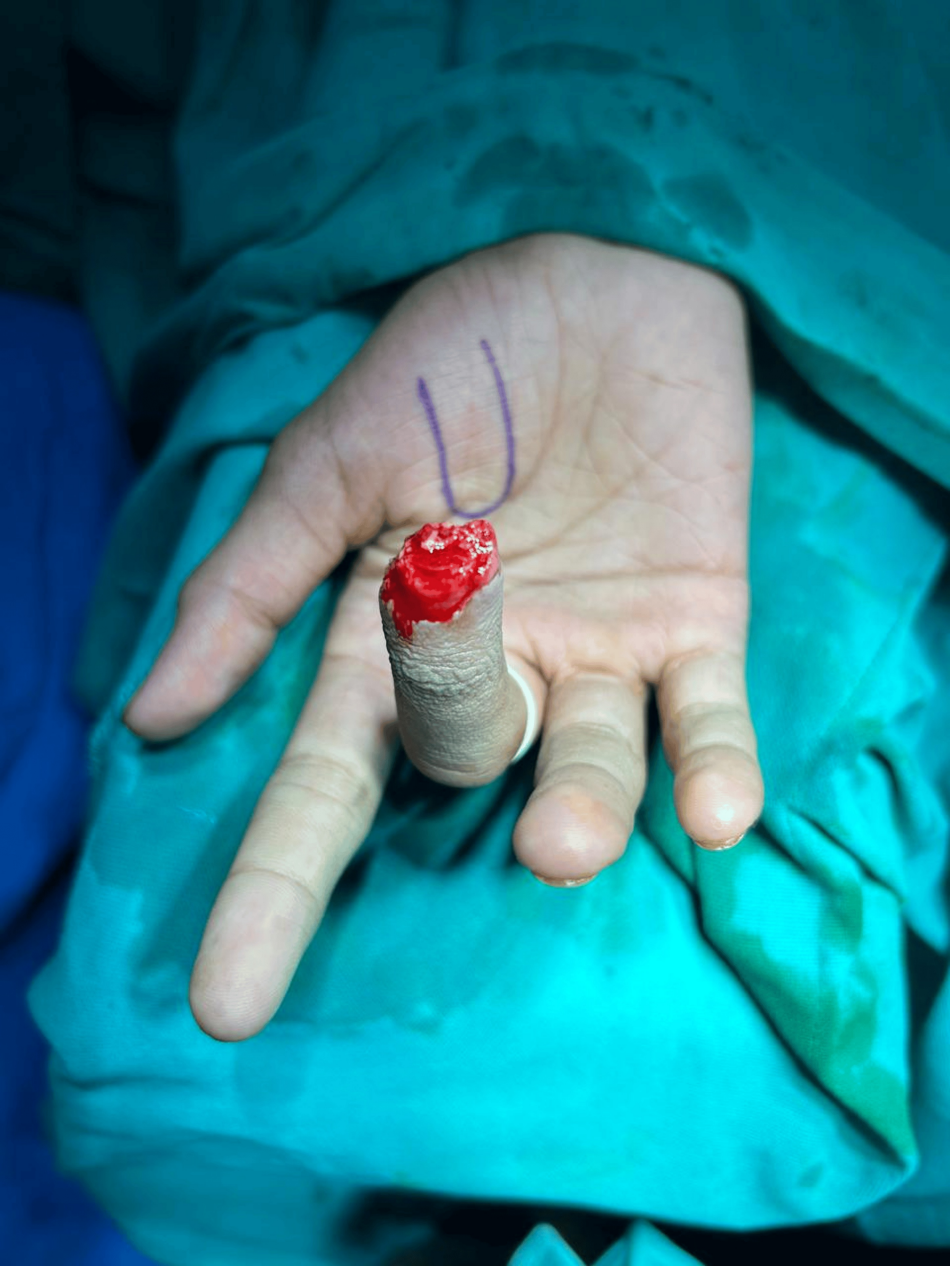
Case 1: Tissue loss noted over tip of distal phalanx of right third digit (top view)

**Figure 3:**
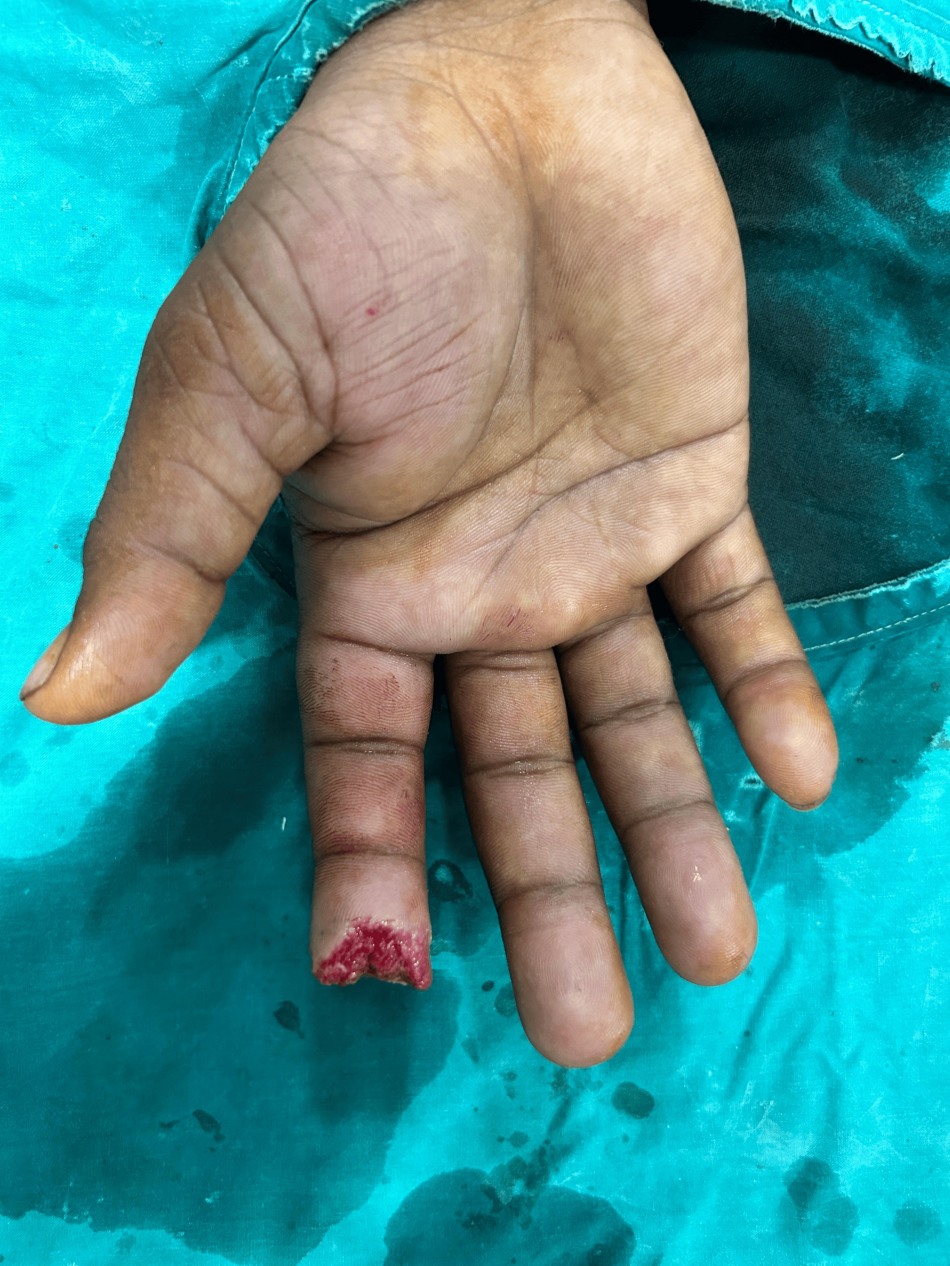
Case 2: Tissue loss with bone loss of distal phalanx of right second digit (palmar aspect)

**Figure 4:**
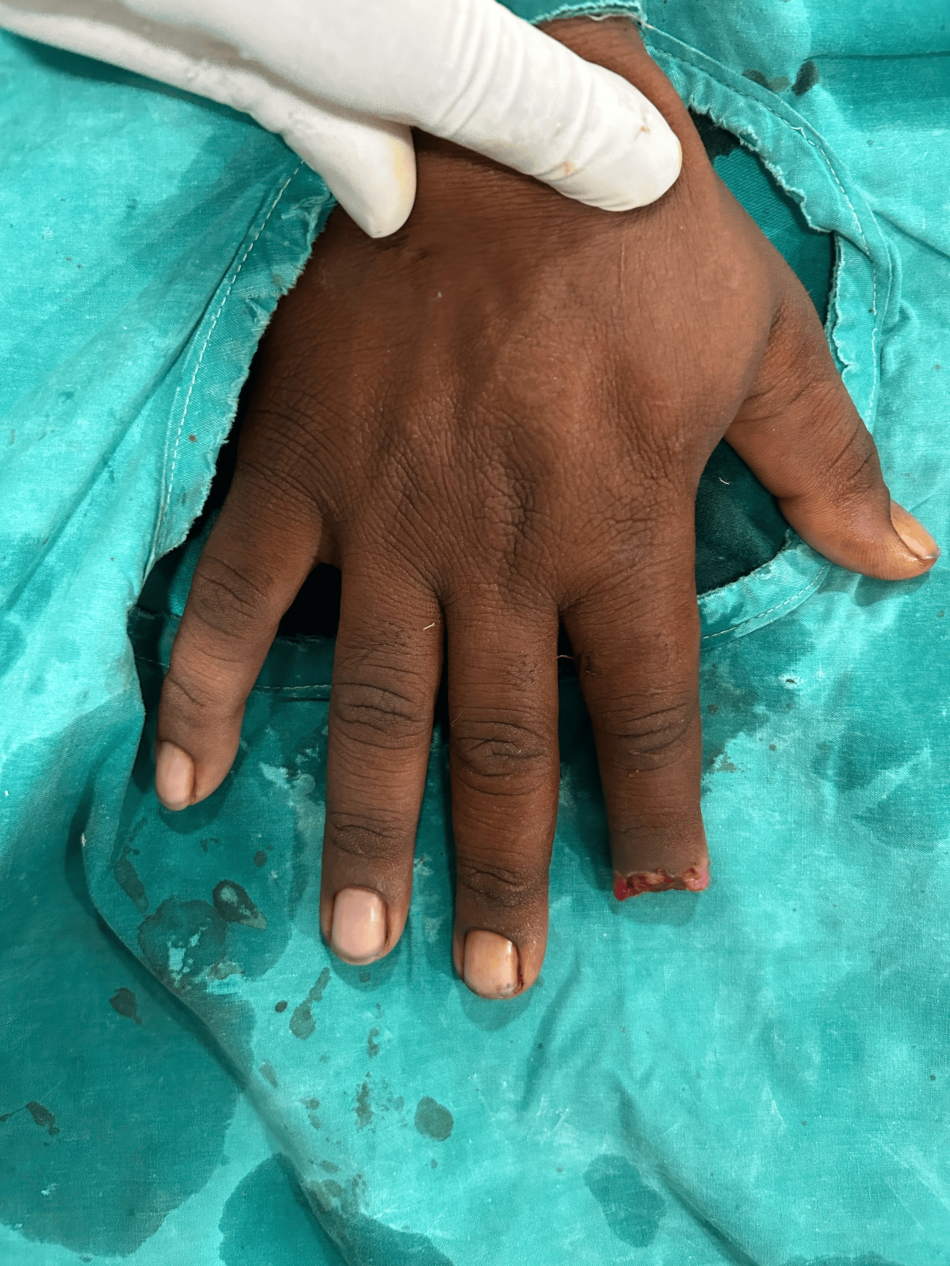
Case 2: Tissue loss with bone loss over right second digit (dorsal aspect)

The majority of cases in this study presented with isolated pulp defects. The design of the thenar flap was tailored to the digit involved. For index finger pulp loss, the flap was raised parallel to the thenar crease, whereas for middle finger defects, it was raised perpendicular or parallel to the thenar crease. In ring finger injuries, the flap was raised perpendicular to the thenar crease. Proximally based flaps were used for transverse defects, while distally based flaps were employed for volar oblique defects. Representative examples of various fingertip injuries are shown in Figures 1-4.

**Figure 5:**
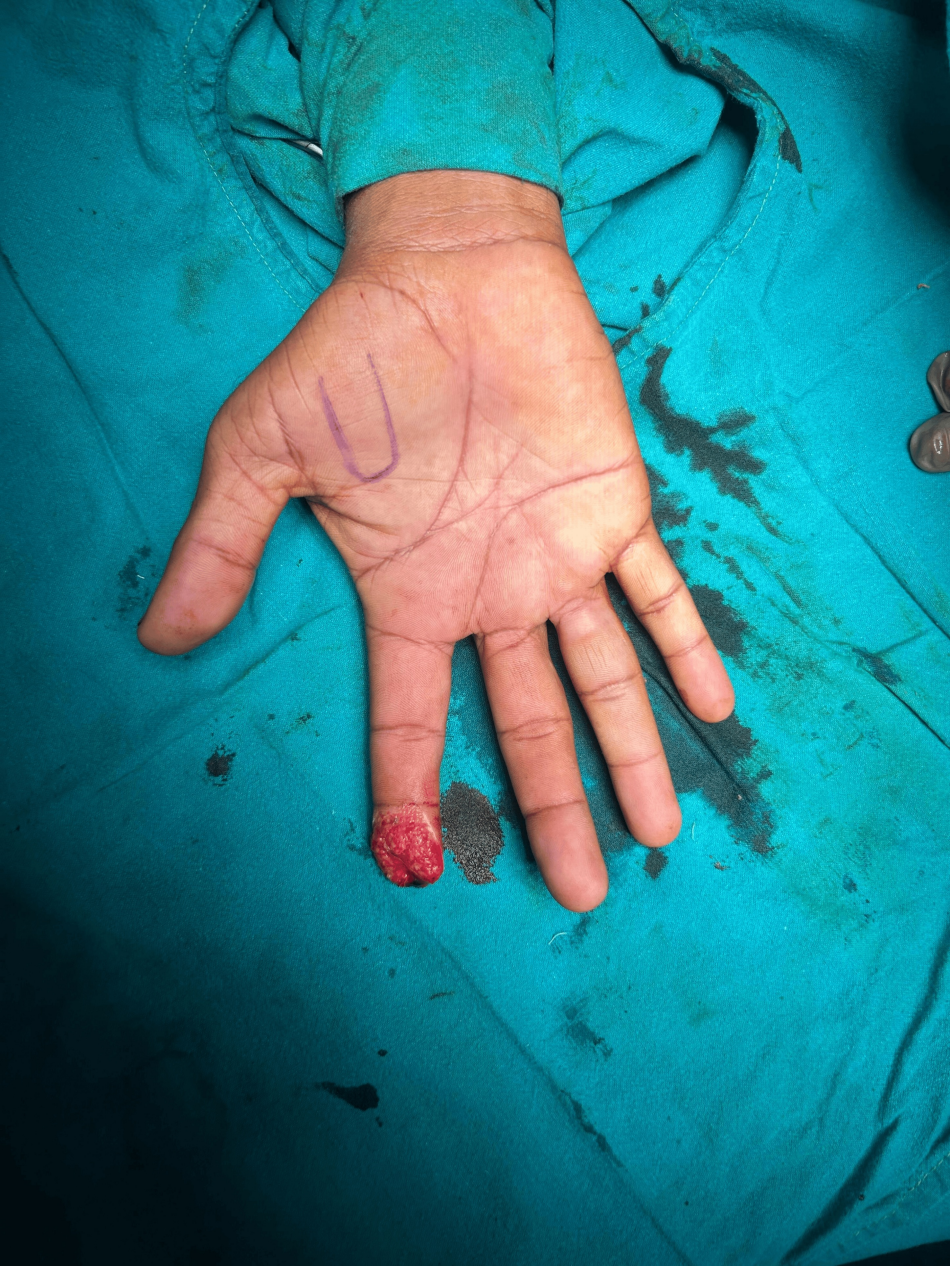
Flap designed parallel to the thenar crease on thenar eminence.

Under regional anesthesia, the flap was raised on the glabrous skin of the thenar eminence, with its proximal edge aligned to the most distal crease of the metacarpophalangeal joint (MCPJ). The flap was elevated in the suprafascial plane, parallel to the thenar crease for index and middle finger injuries and perpendicular to the thenar crease for ring finger injuries. During flap positioning, the MCPJ was flexed to approximately 90°, and the proximal interphalangeal joint (PIPJ) was maintained in slight flexion to minimize the risk of postoperative contracture. The flap dimensions and shape were customized to match the defect, as illustrated in Figure 5.

**Figure 6:**
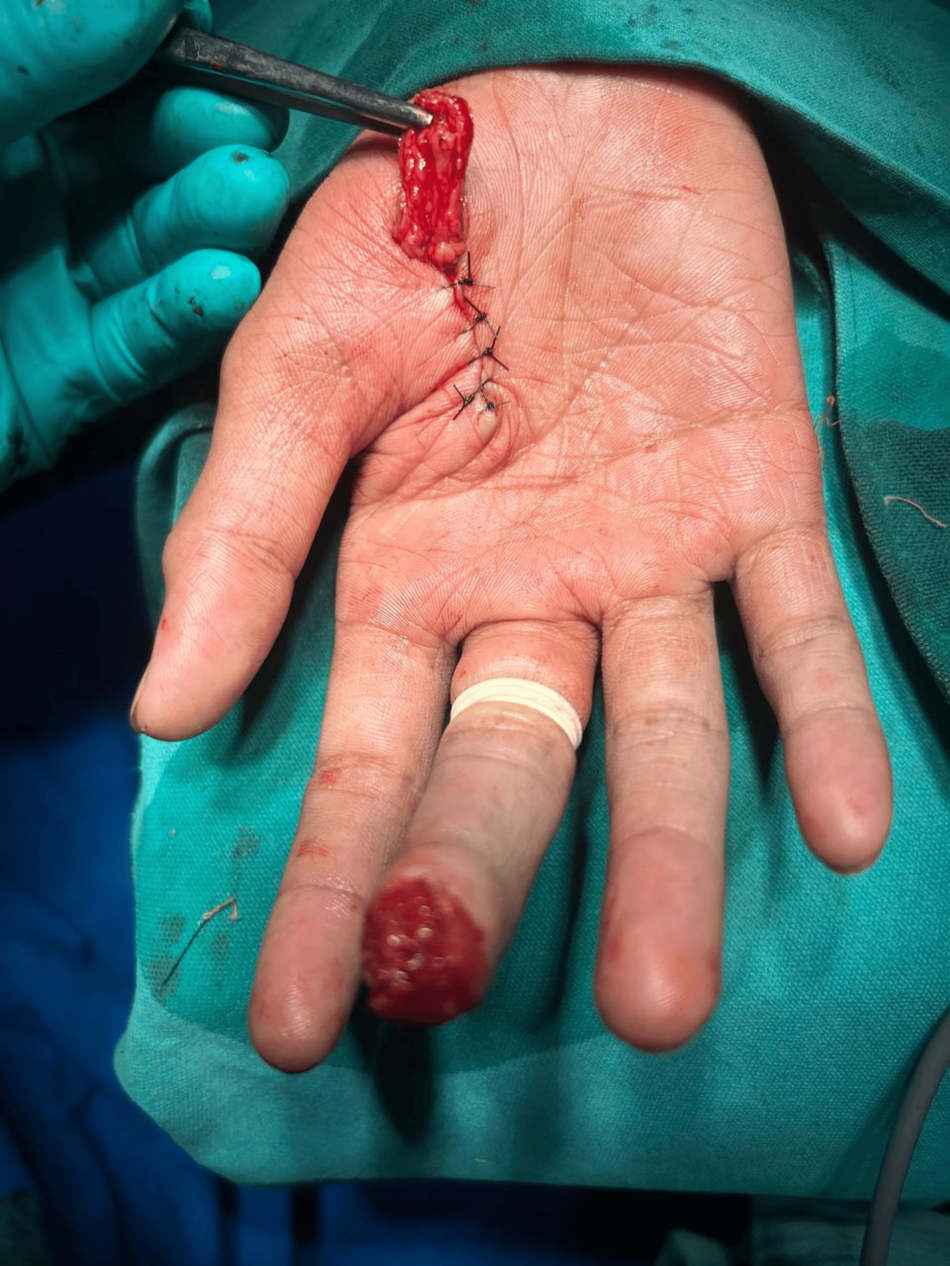
Flap harvested and donor site closed primarily.

Flap dissection was initiated distally and progressed proximally, including subcutaneous tissue and fascia. A subfascial plane was used during dissection to maintain adequate blood supply to the flap. Special care was taken to identify and preserve the radial digital nerve of the thumb. The nerve was not dissected and isolated. The harvested flap is shown in Figure 6.

**Figure 7:**
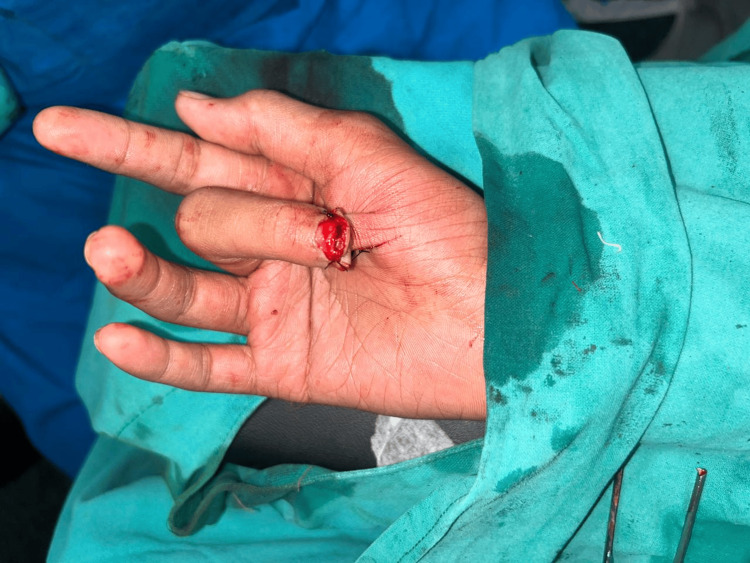
Thenar flap insetting done for middle finger (view from above)

**Figure 8:**
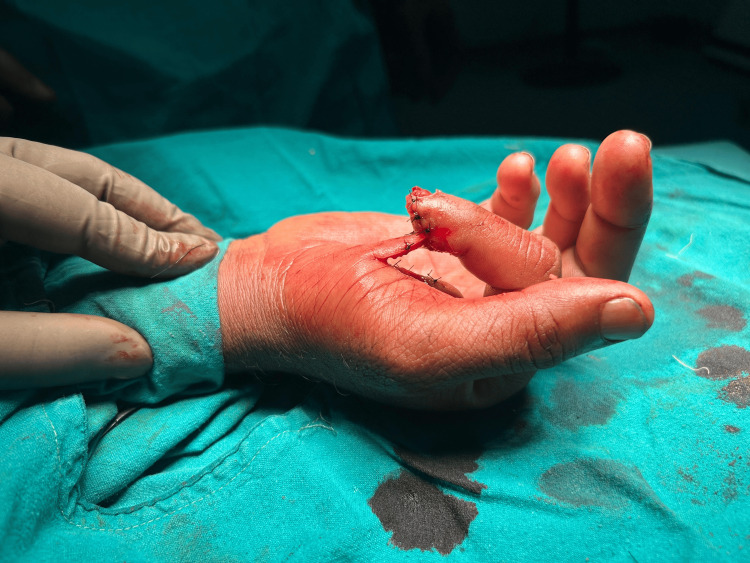
Thenar flap insetting for index finger (view from the side)

The flap was then sutured into place on the injured finger using Prolene 4-0 sutures (Figures 7, 8). Great care was taken to ensure tension-free closure and proper alignment of the flap edges to optimize healing and restore finger function. Hemostasis was confirmed before the dressing was applied.

The affected finger was immobilized in palmar flexion using an elastic adhesive bandage. Patients were instructed to attend follow-up appointments twice weekly for the first three weeks. Flap division and insetting were done at 21 days. The donor site was closed primarily. 

Postop care and follow-up

**Figure 9:**
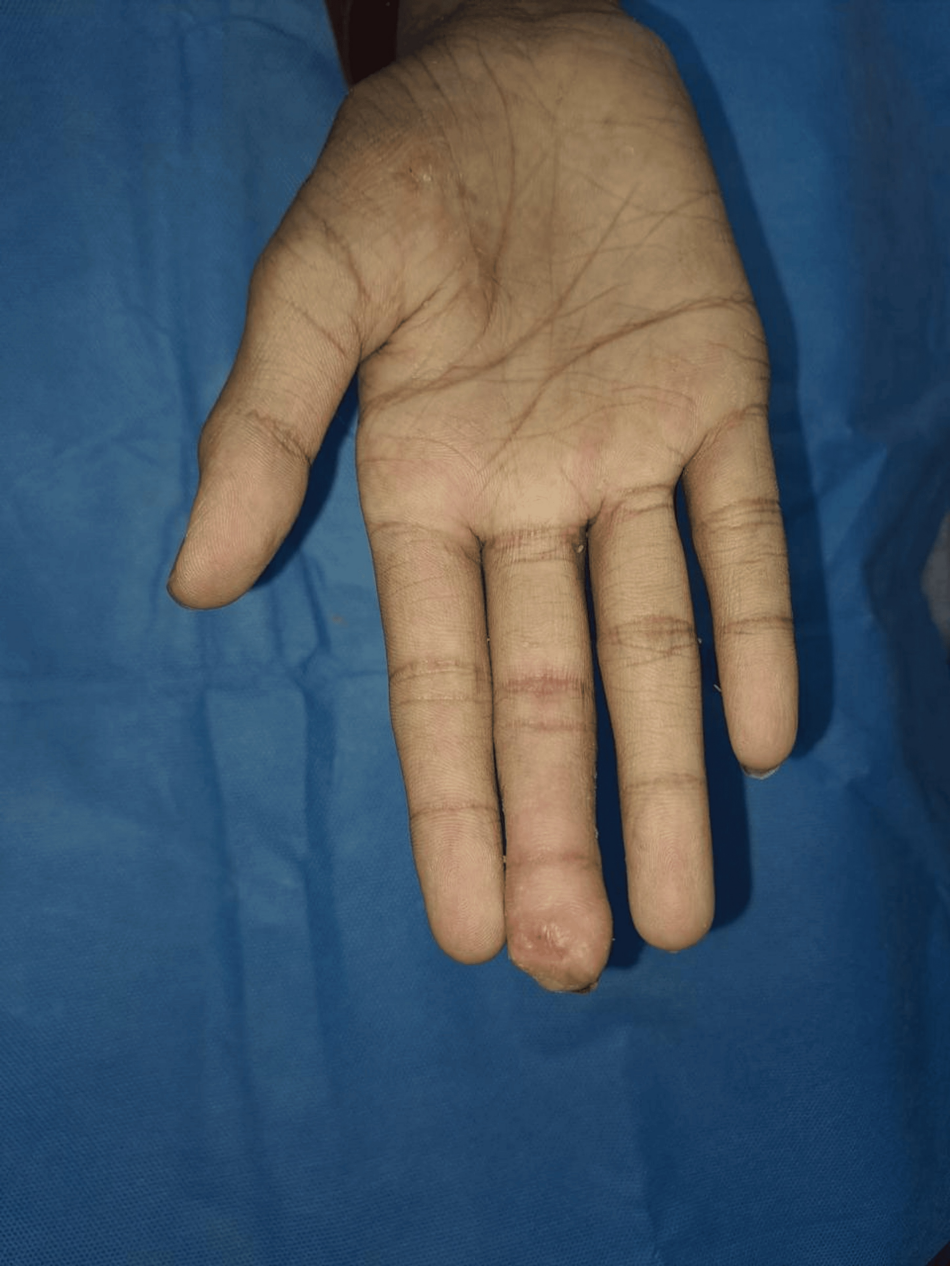
Aesthetic outcome: palmar aspect of right hand

**Figure 10:**
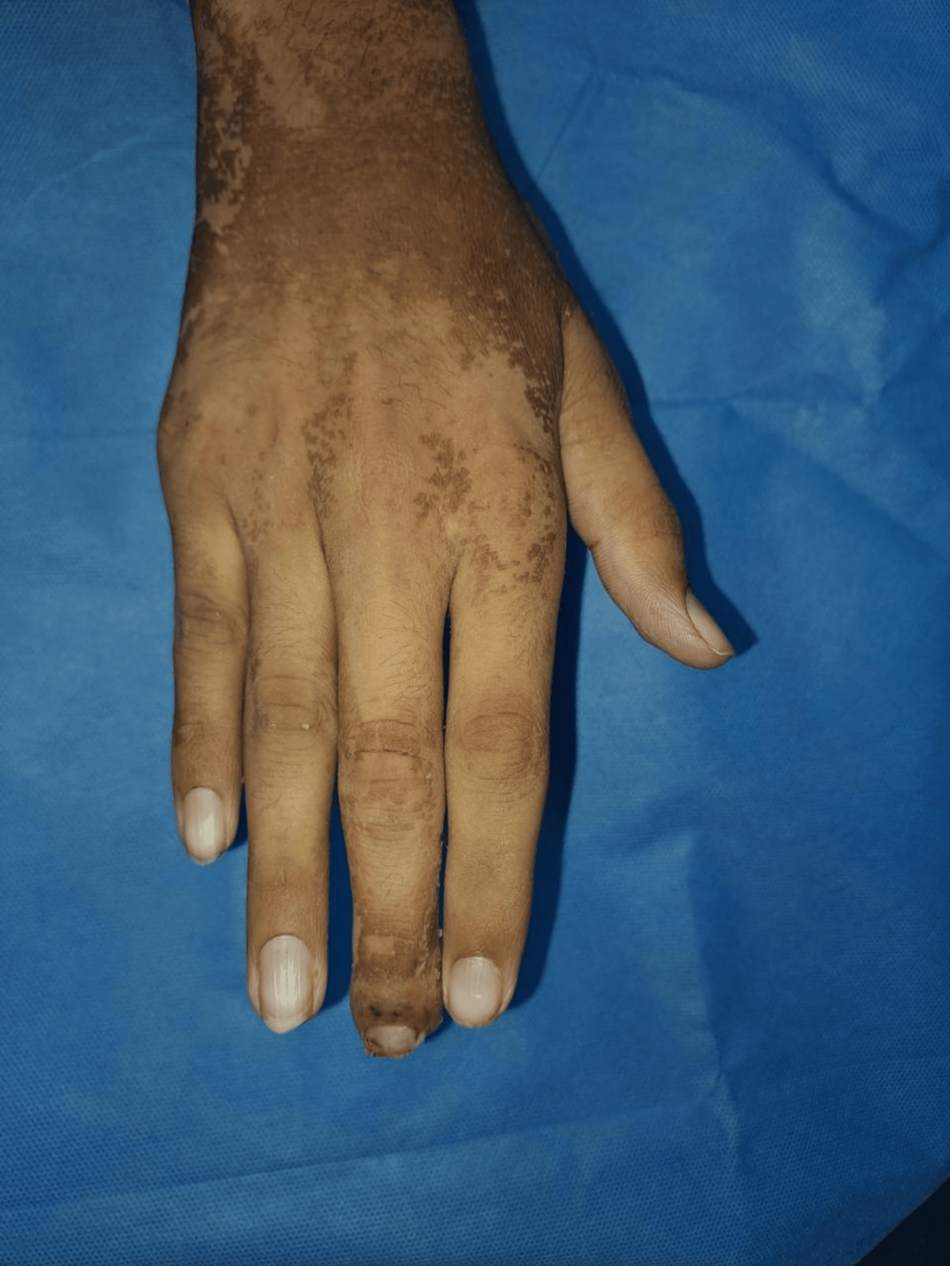
Aesthetic outcome: dorsal aspect of right hand

Patients were followed up twice weekly for the first three weeks to monitor wound healing, flap viability, and early complications. Subsequent follow-ups were conducted at one, three, and six months to assess long-term outcomes such as functional recovery and range of motion (ROM). All patients were prescribed a structured physiotherapy regimen to minimize joint stiffness and enhance rehabilitation of the affected hand (Figures 9, 10).

Outcome measures

Functional outcomes were assessed during follow-ups by measuring two-point discrimination and the ROM at the MP and PIPJ. Subjective outcomes were evaluated using a standardized questionnaire incorporating the satisfaction of the patients over the overall score on a five-point Likert scale, as shown in Table 1. Patients could also submit anonymous comments or suggestions.

**Table 1:** Five-point Likert scale describing patient satisfaction for overall functional and aesthetic outcome

Score	Satisfaction level
1	Very dissatisfied
2	Dissatisfied
3	Neutral
4	Satisfied
5	Very satisfied

## Results

A total of 25 patients with fingertip injuries underwent reconstruction using the thenar flap technique during the study period. This flap was selected due to its reliability, ease of elevation, and suitability for fingertip defects. Details of patient demographics, mechanism of injury, and affected digits are summarised in Table 2. Cases with incomplete follow-up or missing outcome measurements were excluded from statistical calculations for the affected variable.

**Table 2:** Patient data and functional results M: male, F: female, R: right, L: left, F/U: follow-up, MCPJ: metacarpophalangeal joint, PIPJ: proximal interphalangeal joint, ROM: range of motion, 2PD: 2-point discrimination, C/L: contralateral.

S. No.	Sex	Age	Finger	Mechanism of injury	Nature of injury	MCPJ ROM	PIPJ ROM	2PD over reconstructed fingertip	C/L 2PD	Complication	Likert score
1	31	M	R index	Machinery	Volar oblique	99	99	4.2	2	-	5
2	70	F	R middle	Fall of heavy object	Pulp loss	94	90, extensor lag of 10	7.8	3.7	Delayed healing	4
3	24	M	R ring	Stuck in tractor belt	Pulp loss	100	97	4.5	2.8	-	5
4	12	F	R index	Stuck in door	Volar oblique	110	100	4.2	2.2	-	5
5	58	M	L index	Stuck in tractor belt	Pulp loss	96	92, extensor lag of 10	7.4	3.6	-	4
6	46	M	L middle	Machinery injury	Volar oblique	102	97	5.8	2.9	-	5
7	33	F	L index	Machinery injury	Pulp loss	103	95	5.3	2.7	-	5
8	14	M	L index	Stuck in bike chain	Transverse	110	99	4.5	2.1	-	5
9	16	M	R middle	Stuck in tractor belt	Pulp loss	108	97	4.2	2.4	-	5
10	40	M	R index	Fall of heavy object	Transverse	98	96	5.2	3.4	-	5
11	56	M	L ring	Machinery injury	Volar oblique	95	92, extensor lag of 10	6.9	3.5	Distal flap necrosis	4
12	19	M	R middle	Stuck in door	Volar oblique	103	99	4.6	2.8	-	5
13	22	M	R index	Stuck in bike chain	Transverse	105	100	4.3	2.6	Flap detachment	3
14	9	F	R index	Stuck in door	Pulp loss	110	100	4.0	2	-	5
15	23	M	L middle	Stuck in bike chain	Pulp loss	107	96	4.7	2.3	-	5
16	20	F	R middle	Machinery injury	Transverse	108	97	5.0	2.5	-	5
17	23	F	R middle	Machinery injury	Volar oblique	60	70	5.6	2.4	Flexure contracture	2
18	25	M	R index	Stuck in bike chain	Pulp loss	100	99	4.6	2.8	-	5
19	60	M	R index	Fall of heavy object	Volar oblique	96	92, extensor lag of 10	7.2	3.7	Wound dehiscence	4
20	50	M	R middle	Stuck in tractor belt	Transverse	98	94	6.5	3.6	-	5
21	15	M	R index	Stuck in door	Volar oblique	102	99	5.0	2.6	-	5
22	45	M	R index	Fall of heavy object	Pulp loss	99	95	6.8	3.4	-	5
23	65	M	R ring	Fall of heavy object	Volar oblique	95	93, extensor lag of 10	8.0	3.7		4
24	23	M	R index	Stuck in bike chain	Transverse	102	99	5.3	2.8	-	5
25	45	M	R index	Stuck in bike chain	Volar oblique	98	97	5.7	2.5	-	5

Of the 25 patients, 19 were male and 6 were female, with ages ranging from 9 to 70 years and a median age of 25 years. The predominance of male patients reflects the higher exposure to occupational and accidental hand injuries in this demographic. Detailed demographic and injury-related characteristics, including the side of injury, mechanism of trauma, and occupation, are summarized in Table 3.

The majority of injuries occurred in the right hand, 19 cases (76%), and most were due to crush mechanisms, most commonly affecting the index finger (56%), followed by the middle finger (32%) and ring finger (12%). Volar oblique defects accounted for the largest proportion of cases (40%), compared with 24% transverse injuries and 36% pulp loss injuries.

**Table 3:** Demography and injury-related characteristics

Characteristics	n (%)
Number of patients	25 (100%)
Number of males	19 (76%)
Number of females	6 (24%)
Injuries of the right hand	19 (76%)
Injuries to the left hand	6 (24%)
Index finger defects	14 (56%)
Middle finger defects	8 (32%)
Ring finger defects	3 (12%)
Volar oblique injuries	10 (40%)
Transverse injuries	6 (24%)
Pulp loss	9 (36%)

All patients achieved successful reconstruction using the thenar flap without the need for revision surgery. Wounds were managed with meticulous postoperative care, including regular dressing changes, monitoring for signs of infection, and adherence to hand elevation protocols until complete healing was achieved. Dressings were done every 4th day for the first two weeks. Patients were instructed to maintain regular hygiene and to prevent extending the operated finger. Notably, there were no reports of painful or hypertrophic scarring at the donor site, indicating the cosmetic and functional acceptability of the thenar flap technique.

Follow-up

All 25 patients were followed up consistently over six months and evaluated for both functional and aesthetic outcomes. Sensory recovery at the thenar flap donor site was assessed using static two-point discrimination (2PD). At six months, 2PD values ranged from 4.0 to 4.8 with a mean of 5.492. A summary of the results is provided in Table 4. It was observed that older patients tended to exhibit higher (less sensitive) 2PD values, indicating a possible age-related variation in sensory recovery.

**Table 4:** Mean static two-point discrimination test for the affected hand over flap and on same site over contralateral hand

Variables	Mean static 2PD (mm)
Flap	5.492
Contralateral	2.84

Functional evaluation was conducted by measuring the ROM at the MCPJ and PIPJ of the affected fingers. ROM values ranged from 95° to 110° at the MCPJ and 90° to 100° at the PIPJ, with mean values of 99.92° and 95.36°, respectively, as seen in Table 2.

PIPJ stiffness was observed in one patient during the third follow-up visit, approximately two weeks after flap division and insetting (35 days post-initial surgery), with the ROM limited to 70°. This complication was attributed to poor adherence to the prescribed physiotherapy regimen. At the six-month follow-up, the same patient developed a digital flexion contracture and thumb adduction deformity. All other patients achieved full functional recovery by the six-month follow-up after strict compliance with physiotherapy protocols.

All 25 patients completed the postoperative satisfaction questionnaire, which assessed their perceptions of functional and cosmetic outcomes. On the five-point Likert scale, the mean overall satisfaction score was 4.6, indicating a high level of patient satisfaction. In terms of appearance, 88% of patients rated the aesthetic outcome as "satisfied" or "very satisfied," highlighting the cosmetic acceptability of the thenar flap reconstruction.

## Discussion

Fingertip injuries involving volar oblique injuries, significant pulp loss, exposed bone, and transverse injuries pose a complex reconstructive challenge. In such cases, the thenar flap offers a reliable option, utilizing local autologous tissue with sufficient bulk for effective reconstruction and minimal donor site morbidity. The outcomes of the thenar flap are superior in terms of glabrous skin, color match, and sensation. It provides good cushioning to the defect area, which helps patients carry out day-to-day activities.

Historically considered a workhorse flap for fingertip injuries [6-8], the thenar flap had been criticized in earlier literature for potential complications such as flexion contractures, donor site morbidity, and cold intolerance, according to Fitoussi et al. [9]. However, more recent studies, including those on laterally based thenar flaps by Melone et al. [10] and proximally based designs by Barr et al. [11] and Rinker et al. [7], have challenged these concerns, reporting favorable outcomes with improved techniques.

Our findings support the safety and efficacy of the thenar flap when executed with meticulous technique. In our series, the majority of patients who underwent thenar flap reconstruction had better outcomes in terms of cosmetic as well as functional recovery, along with good sensory results. One patient each experienced complications, including delayed wound healing, flap detachment, distal flap necrosis, flexion contracture, and wound dehiscence. Only one patient demonstrated stiffness at the PIPJ at the three-month follow-up, attributed to poor compliance with physiotherapy, and eventually developed flexion contracture.

Consistent with previous literature, distal fingertip injuries in our cohort were more common in males, with more frequent injuries to the right hand, aligning with the population's predominant right-handedness. This observation is similar to that reported by Sahu et al. in 2019 [12].

Sensory recovery of the thenar flap was assessed using static two-point discrimination (2PD) after flap division. Our study reported a mean 2PD of 5.492 mm, which is comparable to previous findings: Dellon (5.6 mm) [13], Barbato et al. (6.5 mm) [14], Sahu et al. (6.33 mm) [12], and Barr et al. (5.5 mm) [11]. These results affirm the consistent sensory performance of the thenar flap across studies.

Functional outcomes were evaluated by measuring the ROM at the MCPJ and PIPJ. In our study, MCPJ ROM ranged from 95° to 110°, and PIPJ ROM from 90° to 100°. These findings are comparable with those reported by Rinker et al. [7], who documented an average MCPJ ROM of 99.58 ± 5.93° and PIP ROM of 90°-110° at one-year follow-up. Similarly, Barr et al. reported an MCPJ ROM of approximately 85° and an average PIP ROM of 103° (95°-110°) [11].

Importantly, no significant differences in aesthetic or functional outcomes were observed across different age groups in our study. These findings reinforce the reliability and versatility of the thenar flap technique, particularly when combined with proper flap planning, precise surgical technique, and adherence to postoperative rehabilitation.

The thenar flap offers a three-dimensional, composite reconstruction that is both functionally and aesthetically satisfactory while preserving sensation over the thenar region. This is supported by our patient questionnaire results, in which 88% of respondents rated outcomes as "satisfied" or "very satisfied." Similarly, Rinker et al. [7] reported that 75% of their patients rated function, durability, and aesthetic appearance as good or excellent.

Only one patient developed flexion contracture, while none had sensory loss or disfigurement, consistent with other studies. Therefore, the thenar flap remains a superior option for fingertip reconstruction when proper flap design principles, meticulous wound care, and early mobilization are employed, resulting in excellent clinical outcomes.

Limitations of this study include a relatively small sample size and the absence of a comparative analysis with alternative flap techniques. Due to the small sample size, the results were more biased toward the thenar flap. Another limitation of the present study is the lack of comparison with newer reconstructive techniques, such as microvascular tissue transfer and technically demanding flaps, which may provide alternative outcomes in contemporary practice. The focus of this study, however, was to re-establish the utility of the thenar flap in general practice and resource-restricted settings where such advanced options may not be readily available.

While newer reconstructive options such as microvascular tissue transfer and technically demanding local flaps are available, the thenar flap offers several distinct advantages. It is technically straightforward, does not require microsurgical expertise, and can be performed in resource-limited settings without specialized infrastructure. The procedure is reliable, provides good soft tissue coverage with satisfactory cosmetic and functional outcomes, and avoids the morbidity and prolonged operative time associated with complex microvascular techniques. These advantages reaffirm the thenar flap as a practical and valuable option in fingertip reconstruction, particularly in general practice and in regions where access to advanced reconstructive methods is limited.

Furthermore, surgeon expertise and patient compliance with postoperative physiotherapy, both of which varied, may have acted as confounding factors. Future studies with larger randomized cohorts and standardized rehabilitation protocols are needed to validate these results. Nonetheless, we conclude that the thenar flap is a safe and effective reconstructive choice for fingertip injuries across all age groups, providing adequate tissue bulk and color match with minimal morbidity.

## Conclusions

The thenar flap remains a reliable and versatile option for fingertip reconstruction, particularly for volar oblique and transverse injuries associated with pulp loss and exposed structures. When executed with meticulous surgical technique and followed by a structured physiotherapy regimen, it offers excellent functional recovery, minimal donor site morbidity, and high patient satisfaction. Its technical simplicity and reproducibility make it especially suitable for general practitioners in peripheral and rural settings.

This study reinforces the utility of the thenar flap across a broad age spectrum, challenging earlier perceptions that limited its application in older patients. Although minor complications were noted, the overall success rate and favorable aesthetic outcomes support its role as a workhorse flap in fingertip reconstruction. Future prospective studies incorporating direct comparisons with alternative flap techniques would strengthen the evidence base. Additionally, further research into fascial flap methods, in which adipofascial tissue is transferred to the defect site, may help reduce donor site morbidity while maintaining optimal reconstructive outcomes.
